# Material Evaluation and Process Optimization of CNT-Coated Polymer Powders for Selective Laser Sintering

**DOI:** 10.3390/polym8100370

**Published:** 2016-10-19

**Authors:** Shangqin Yuan, Jiaming Bai, Chee Kai Chua, Jun Wei, Kun Zhou

**Affiliations:** 1 Singapore Centre for 3D Printing, School of Mechanical and Aerospace Engineering, Nanyang Technological University, 639798 Singapore, Singapore; yuan0057@e.ntu.edu.sg; 2 Singapore Institute of Manufacturing Technology, 638075 Singapore, Singapore; baijm@SIMTech.a-star.edu.sg (J.B.); jwei@simtech.a-star.edu.sg (J.W.)

**Keywords:** selective laser sintering, carbon nanotubes-reinforced nanocomposite, polyamide 12, additive manufacturing, 3D printing

## Abstract

Multi-walled carbon nanotubes (CNTs) as nano-reinforcements were introduced to facilitate the laser sintering process and enhance the thermal and mechanical properties of polymeric composites. A dual experimental-theoretical method was proposed to evaluate the processability and predict the process parameters of newly developed CNT-coated polyamide 12 (CNTs/PA12) powders. The thermal conductivity, melt viscosity, phase transition and temperature-dependent density and heat capacity of PA12 and CNTs/PA12 powders were characterized for material evaluation. The composite powders exhibited improved heat conduction and heat absorption compared with virgin polymer powders, and the stable sintering range of composite powders was extended and found to be favourable for the sintering process. The microstructures of sintered composites revealed that the CNTs remained at the powder boundaries and formed network architectures, which instantaneously induced the significant enhancements in tensile strength, elongation at break and toughness without sacrificing tensile modulus.

## 1. Introduction

Additive manufacturing (AM) techniques are a collection of advanced layer-by-layer processes which produce physical three-dimensional (3D) objects directly from the pre-programmed and sliced 3D model data through an additive approach [1,2,3]. As opposed to the conventional subtractive processes cutting away materials from a big workpiece or moulding processes, AM offers great flexibility with respect to product design and manufacture [1].

Laser sintering, or selective laser sintering (SLS), is one of the most established powder-based AM processes [4,5,6]. The principal advantage of SLS is the ability to create complex geometries, compared with the conventional processes, such as cutting, tooling and moulding. Additionally, no supporting materials are required as the powder-bed itself acts as a supporting structure that can easily be removed. Thus, the cost for the post-process of SLS is greatly lower than that of other types of AM techniques, such as selective laser melting [7,8], stereolithography and inkjet printing [9,10,11]. Theoretically, a large number of thermoplastic powders can be enabled to be processed using laser sintering.

However, few polymers are commercially available for laser sintering, whereas hundreds of grades are available for conventional manufacturing techniques, such as injection moulding [12,13]. Many thermoplastics show a limited processability in laser sintering, resulting in sintered parts with inferior mechanical and thermal properties. Furthermore, the available polymers, such as polyamides (PAs), polyethylene, polycarbonate and polyurethanes, cannot completely meet the needs of industrial applications due to the unsatisfied mechanical, thermal orelectrical properties [4,14].

Due to the current limited choices of single polymers for laser sintering and the inferior properties of sintered polymer parts, filler/polymer composites have been investigated to enhance the mechanical, thermal or electrical properties [15,16]. Bai et al. investigated a novel method to prepare CNTs-functionalized PA12 nanocomposite powders by coating CNTs onto the surface of PA12 powder particles [17,18]. This allowed the optimized size and near-spherical morphology of the commercial PA12 powders to be retained. The sintered CNTs/PA12 exhibited the slightly improved thermal conductivity, which is capable of facilitating the laser sintering process [19]. However, in spite of thermal conductivity, CNTs/polymer nanocomposite powders exhibited different manners, especially thermal and dynamic mechanical behaviours. These changes might simultaneously influence the sinterability and process parameters of materials and affect the performances of the sintered parts [20,21].

The determination of processing parameters for a newly developed polymer or polymeric composite to achieve desirable part performance is still challenging in the SLS system because laser sintering is a complex thermal and dynamic process that includes heat absorption, heat transfer, phase change and melt flow manners [19,21]. The empirical optimization of processing parameters is based on an iteration of trial and error builds, which is highly time-consuming and extremely expensive depending on the cost per unit of materials, especially for non-recyclable polymers. Recently, Vasquez et al. [22] and Berretta et al. [20] employed a theoretical model to predict the process parameters through calculating the thermal energy to melt of applied powders. The influencing factors, such as the bed temperature, powder packing density, specific heat, melt temperature and heat of fusion, were taken into consideration. A stable sintering region (SSR) was introduced to describe the optimum temperature range for a successful sintering process. The evaluation of the energy absorption within this range was helpful to determine the reasonable processing parameters [22]. The polymer powders were melted to coalesce in their liquid state and then recrystallized to form solid structures upon the superfast heating and cooling cycle. The intrinsic properties of the polymer powders exhibited temperature and phase-dependent behaviours, whereas the influences of these changes on laser sintering process are poorly understood, and few experimental studies can quantitatively apply the exo- and endothermal energy to predict process parameters.

In this work, a novel method was proposed to predict the laser energy input range and then narrow the working range of laser parameters to effectively optimize sintering. Together, the reinforcement mechanism of CNTs within sintered PA12 matrix was investigated through analysing the thermal influences of addition of CNTs, the microstructures and mechanical properties of the sintered composite parts. The heat capacity and density in the powder, melting and liquid phases were characterized and implemented to the theoretical model to predict the energy required for melting and decomposition. The laser absorbed by powders should be within the energy range from the melting to decomposition of PA12 or CNTs/PA12. This method of material evaluation and process optimization is cost effective and generally applicable for new material development in the SLS system.

## 2. Materials and Methods

### 2.1. Material Preparation

Commercially available thermoplastic PA12 powders (PA2200 from EOS GmbH, Munich, Germany) were the polymer material used in the SLS system. The aqueous solution of CNTs was 3 wt % with the purity greater than 95% (purchased from Nano Amor Materials Inc., Houston, TX, USA). The dimensional parameters of these CNTs were 20–30 nm in diameter and 10–30 µm in length. Sodium cholate hydrate (from ox or sheep bile with the purity greater than 99%, purchased from SIGMA Life Science, Singapore) was applied to functionalize CNTs and facilitate their assembly on the surfaces of the polymer powders.

PA12 powders with a thin layer of CNTs-coating were obtained through a surfactant-facilitated latex method as described by Cai et al. [23]. The steroid-based surfactants were used to modify CNTs in an aqueous solution, and then this solution was mixed with the water suspension of polymer powders to coat CNTs into the polymer powders at an elevated temperature. Powders were precipitated and filtrated to dry within a proper range of temperature. The nanocomposite powders could maintain initial shape, size and size distribution of original powders coated with CNTs. In this work, the addition of CNTs for this powder composite was with a weight fraction of 0.5%. Afterwards, the residual solvent absorbed by the polymer was evaporated through further drying in an oven at 70 °C. The detailed composition of CNTs and PA12 and the preparation process were reported in our previous work [24].

### 2.2. Material Characterization and Numerical Evaluation

**Figure 1:**
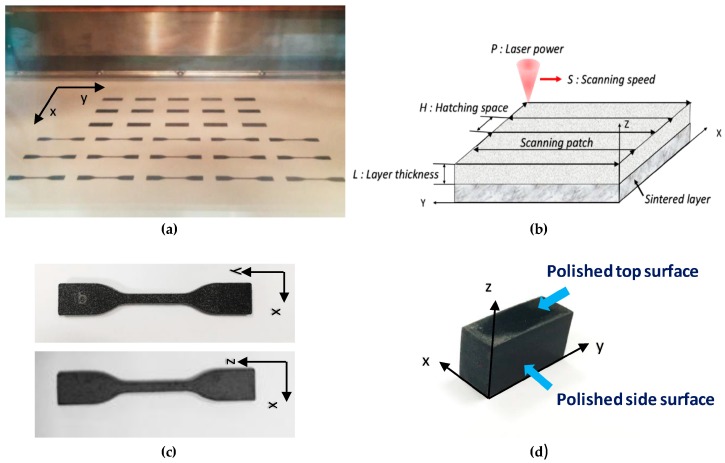
(**a**) building platform of EOS P395 machine regarding as the *X*–*Y* plane; (**b**) schematic illustration of the critical parameters in laser sintering process; (**c**) micro tensile specimens (ASTM D638, type IV) produced in the *X*–*Y* and *X*–*Z* planes; (**d**) the polished specimen for microstructure characterization in the *X*–*Y* (top surface) and *Y*–*Z* (side surface) planes.

A field emission scanning electron microscope (FESEM) (JSM-7600F, JEOL Ltd., Tokyo, Japan) was used to characterize the surface morphologies of the polymeric nanocomposite powders with the e-beam power of 2–5 KV. In order to capture the network structure of CNTs within the polymer matrix after sintering, the optical microscopy (Olympus DP72, Southborough, MA, USA) was employed to investigate the microstructures of the sintered CNTs/PA12 composites, which were properly ground and polished to achieve smooth and reflective surfaces as shown in Figure 1d. The flowability of polymer powders was quantified through the flowability measurement conducted by Revolution Powder Analyser (Basel, Switzerland), based on evaluated process conditions. The sample powders were placed inside the measurement drum. The instrument was run for 250 sets of avalanches or data points.

During the transitions of solid powder-melting metaphase-liquid phase, the critical thermal properties of the polymer powders were changed significantly. For instance, the changes of the heat capacity, density and melt viscosity of the polymer powders strongly influenced their heat absorption, melting and coalescence over the entire sintering process. Therefore, the concepts of modified heat capacity and density were introduced to quantitatively evaluate the processability of PA12 and CNTs/PA12 in laser sintering. The experimental-theoretical method was utilized to fit the experimental results of temperature-dependent properties at different states and then implement these functions to predict the energy absorption and consumption during sintering. Thus, the portable working range of the sintering parameters could be determined.

Differential scanning calorimetry (DSC) was carried out using a Q200 DSC from TA Instruments (New Castle, DE, USA) under an air atmosphere. The PA12 and CNTs/PA12 powders with the mass of 5 mg were encapsulated in a TA Tzero standard aluminum pan and loaded into DSC for heating-cooling loop testing. The thermal program for this experiment was as follows: cool rapidly to T = 25 °C, hold isothermally for 1 min, heat up with a rate of 10 °C/min to 250 °C, hold isothermally for 1 min, cool to T = 25 °C with a rate of 10 °C/min, hold isothermally and finally stop. This heating-cooling programming was conducted to identify the onset and offset of melting and recrystallization as well as the enthalpy changes during phase transitions. In addition, a modulated model of DSC was applied to characterize the modified heat capacity of PA12 and CNTs/PA12 from powders to melt manners following the ASTM standard E1269-11 [23] and the modified heat capacities with respect to the three stages of powder phase, melting metaphase and liquid phase were calculated by Equations (1)–(3) [24]

(1) $$C_{P}^{*}=C_{P}^{P}\left(T\right),\;T<T_{\mathrm{ms}}$$

(2) $$C_{P}^{*}=C_{P}^{P}\left(T_{ms}\right)+\frac{\Delta H_{m}}{\sqrt{\pi {\left(T_{mf}-T_{ms}\right)}^{2}}}\exp\left(-\frac{{\left(T-T_{ms}\right)}^{2}}{{\left(T_{mf}-T_{ms}\right)}^{2}}\right),\;T_{\mathrm{ms}}<T<T_{\mathrm{mf}}$$

(3) $$C_{P}^{*}=C_{P}^{m}\left(T\right),\;T>T_{\mathrm{mf}}$$

where *T*_ms_ and *T*_mf_ are the onset and offset of melting, respectively. With the three stages from powder to metastable melting phase to fully melted phase, $C_{P}^{P}\left(T\right)$ is the heat capacity of the material in its powder form, which considers the influence of empty spaces among free-compacted powders; $C_{P}^{m}\left(T\right)$ is the heat capacity of material in a liquid phase. Additionally, $\Delta H_{m}$ is the melting enthalpy.

The volumetric changes of materials upon melting could be characterized through a novel method developed by Verbelen et al. [19]. The basic procedures of the method were: (i) to build a special container and adapt it into a thermomechanical analyzer (TMA) (Q400, TA instruments, New Castle, DE, USA); (ii) to disperse polymer or its composite powders into silicon oil and fill this mixture into the confined container; (iii) to measure the difference of volume expansion between pure silicon oil and the suspension of powders. The temperature-dependent specific volume of the polymer powders could be measured at a heating rate of 0.5 °C/min. The instant density of the polymer powders was regarded as the reciprocal of the specific volume of material. Thus, the modified densities $\rho^{*}\left(T\right)$ of PA12 and CNTs/PA12 were temperature-dependent functions within the different stages of heating and could be obtained by

(4) $$\rho^{*}\left(T\right)=\rho_{p}\left(T\right)=\frac{1}{V_{p}\left(T\right)},\;T<T_{\mathrm{ms}}$$

(5) $$\rho^{*}\left(T\right)=\rho_{m}\left(T\right)=\frac{1}{V_{m}\left(T\right)},\;T_{\mathrm{ms}}<T<T_{\mathrm{mf}}$$

(6) $$\rho^{*}\left(T\right)=\rho_{l}\left(T\right)=\frac{1}{V_{l}\left(T\right)},\;T>T_{\mathrm{mf}}$$

where $\rho_{p}\left(T\right)$ and $V_{p}\left(T\right)$ are the modified density and specific volume of powder form, respectively. $\rho_{m}\left(T\right)$ and $V_{m}\left(T\right)$ are these of the melting metaphase, and $\rho_{l}\left(T\right)$ and $V_{l}\left(T\right)$ are these of the liquid phase.

Thermogravimetric analysis (TGA) was conducted using TGA Q500 equipment (TA instrument, New Castle, DE, USA). For each run, the TGA results were acquired from approximately 15 mg of PA12 and CNTs/PA12 powders, which were heated from the room temperature up to 650 °C with the rate of 10 °C/min under the N_2_ gas environment, in order to obtain the decomposition profiles of the specimens at an elevated temperature.

Rheological dynamic measurements were performed at 200 °C in the strain-controlled DHR-Rheometer (TA-Instruments, New Castle, DE, USA), using the 25 mm diameter parallel plates. The powders were compressed into a cylinder (diameter in 25 mm and thickness in 2 mm) by mechanical force. The specimens were tested in an oscillating dynamic mode, the frequency was varied from 100 to 0.1 rad/s, and the melt viscosities of PA12 and CNTs/PA12 were characterized.

### 2.3. Selective Laser Sintering

The SLS machine (EOS P395 model from EOS GmbH, Munich, Germany) was used to conduct the laser sintering process of PA12 and CNTs/PA12 powders. The CO_2_ laser with a wavelength range of 1060 um can generate continuous laser up to 50 W. A commercialized set of parameters for PA12 processed by the EOS P395 machine is listed in Table 1. The set of industrial parameters was implemented to the sintering process of PA12 and CNTs/PA12 powders in this research, in order to compare the different performances of the sintered parts and investigate the influences of CNTs on the polymer powder-based sintering.

To further investigate the relationship between process–structure–properties in the laser sintered CNTs/PA12 products, the critical parameters such as the laser power *p*, scanning speed *s* and hatch space *h* were tailored and separately set in five discrete levels as listed in Table 2. The energy input per unit area *E_area_* and the volume *E*_vol_ from the laser are determined by

(7) $$E_{area}=p/\left(h\;s\right)$$

(8) $$E_{vol}=\frac{p}{h\;s\;L}$$

where *L* is the layer thickness (Figure 1b).

The energy per volume required for melting $E_{mv}$ is defined

(9) $$E_{mv}=\rho^{*}\left(T\right)\phi \int_{T_{b}}^{T}C_{p}^{*}dT,\;T<T_{\mathrm{mf}}$$

The energy absorption per volume $E_{dv}$ within the stable sintering region (SSR) is described by

(10) $$E_{dv}=\rho_{l}\left(T\right)\int_{T_{mf}}^{T_{ds}}C_{P}^{m}dT,\;T_{mf}<T<T_{ds}$$

SSR describes the optimum temperature range for successful laser sintering usually from the offset of melting $T_{mf}$ to the onset of decomposition $T_{ds}$ where indicates the 1% weight loss from TGA diagram [22].

The substitution of the modified density $\rho^{*}\left(T\right)$ into Equation (9) results in

(11) $$E_{mv}=\rho_{p}\left(T\right)\phi \int_{T_{b}}^{T_{ms}}C_{P}^{P}dT+\rho_{m}\left(T\right)\int_{T_{ms}}^{T_{mf}}C_{P}^{*}\;dT,\;T<T_{mf}$$

where ϕ is the packing factor of the polymer powders, $T_{b}$ is the bed temperature of laser sintering. The modified density and heat capacity are functions of temperature and they will be used to predict the energy absorptions according to Equations (9) and (11).

In order to effectively sinter polymer powders and prevent polymer degradation or decomposition, the input laser energy should satisfy a relationship expressed as

(12) $$E_{mv}<E_{vol\;}\alpha_{critical}<E_{dv}$$

where $E_{vol}$ is the laser energy per volume; $\alpha_{critical}$ is the critical heat absorptivity of polymer powders exposed to laser heat. This relationship can be applied to evaluate the heat absorptivity incorporating experimental observations, and employed to predict the proper process parameters that could induce sufficient energy for melting and keeping the heating temperature within the stable sintering region (SSR).

Figure 1a demonstrates the building bed (*X*–*Y* plane) for sintering CNTs/PA12 indicating that composite powders were immediately darkened and formed solid bulky composites. The critical process factors influencing energy input of laser in the specific volume are illustrated in Figure 1b. The specimens were printed in the *X*–*Y* and *X*–*Z* planes in order to investigate the anisotropic tensile properties and microstructures induced by the layer-by-layer powder sintering process. Dog-bone-shaped samples (refer to Figure 1c, which follows the ASTM D638 standard, type IV) were prepared for uniaxial tension loading by using an Instron (Norwood, MA, USA) 5565 testing system. Five samples were printed with respect to each set of parameters for the tensile testing.

## 3. Results and Discussion

### 3.1. Evaluation of CNTs/PA12 for SLS Compared with PA12

**Figure 2:**
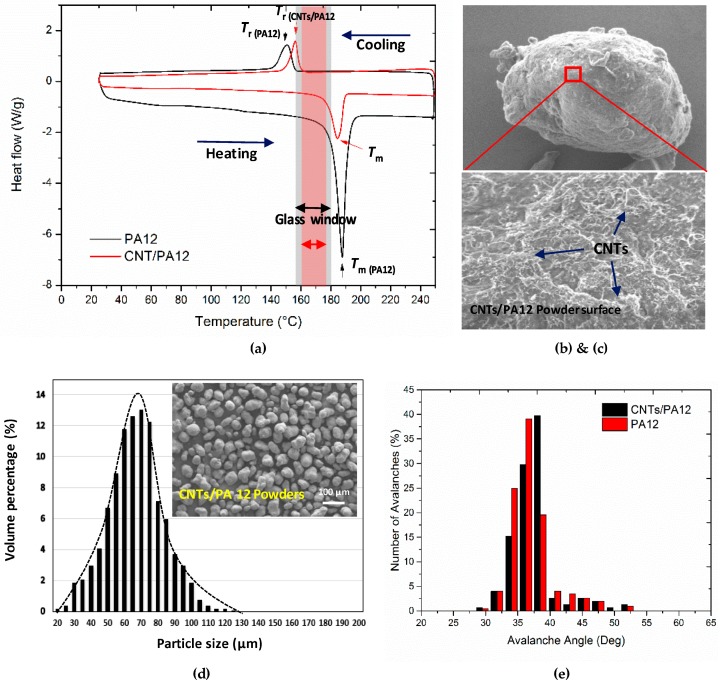
(**a**) DSC diagrams of PA12 and CNTs/PA12 upon heating and cooling at the rate of 10 °C/min; (**b**) size distribution of PA12 and CNTs/PA12 (0.5 wt %); SEM images of (**c**) the SEM image of an entire composite powder and then zoomed in to investigate the surface coating of CNTs; (**d**) the surface layer of CNTs which are lighten network structure; (**e**) avalanche angle graph indicating the required average angle to start and maintain the flow of the PA12 and CNTs/PA12 powders.

The size and morphology of composite powders were investigated (Figure 2), and the particle size was mainly distributed within a narrow range of 60–70 µm, which is consistent with that of the neat PA12 (PA2200) as reported in the literature [25,26]. This is because CNTs’ coating is extremely thin as compared with the diameter of the polymeric powders. The original near-spherical shape of the polymer powders was retained and such shape and morphology of these powders exhibited proper flowability and were preferable in the layer-by-layer deposition [18,21]. Besides, CNTs were able to assemble uniformly onto the surface of polymer powders and form a network structure as shown in Figure 2d.

The melting and recrystallization of materials were studied using DSC. The melting peaks provide the important indications on sinterability of materials. PA12 shows a very sharp melting peak with high enthalpy of fusion because of the high crystal perfection of virgin powders [21]. It also indicates that the energy trigger of melting is required to be strong and fast; this is just the unique advantage of laser energy. In comparison, CNTs/PA12 shows a relatively smooth peak and a low melting point, which indicates the improved heat conductance and efficiency of fusion. This observation implies that the requirement of thermal energy input from laser can be reduced and the full melting of CNT-introduced composite can be easy to achieve.

The recrystallization peak of CNTs/PA12 is relatively sharp and narrow compared with that of PA12, because the CNTs can act as a nucleating agent and accelerate the crystallization process [25]. However, the onset of recrystallization of PA12 is at a lower temperature point than that of its composite and the broad peak of PA12 indicates its gradual crystallization kinetics.

In general, the process temperatures such as pre-heating temperature (*T*_b_) and chamber temperature (*T*_c_) are roughly set based on the DSC heating-cooling cycle. The glass window is within the temperature range of melting and crystallization onsets. Usually, the *T*_b_ should be set within the glass window and just below the melting onset. *T*_c_ is set below the recrystallization offset. Since the polymers for the laser sintering process experience the transitions from powder form to liquid phase to solid form, the critical temperature transitions are crucial for quantitatively evaluating the energy input for heating or melting. In this study, as illustrated in Figure 2a, the glass window of CNTs/PA12 was evaluated by DSC analysis and it is narrower than that of PA12. DSC heating-cooling curves were also utilized to obtain the onsets, offsets and absolute enthalpy values of melting and recrystallization of PA12 and CNTs/PA12 for further quantitative evaluation (Table 3).

**Figure 3:**
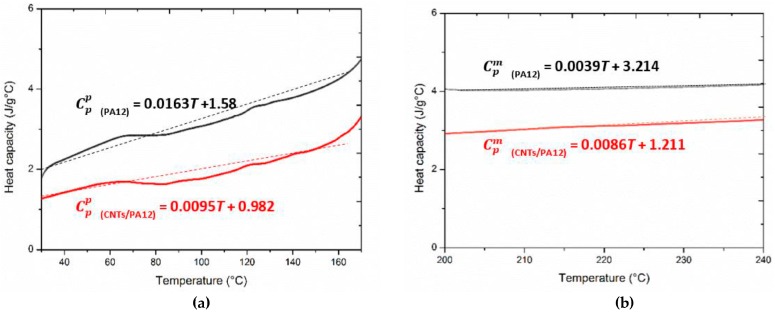
Specific heat of PA12 and CNTs/PA12 at (**a**) powder phase and (**b**) liquid phase.

**Figure 4:**
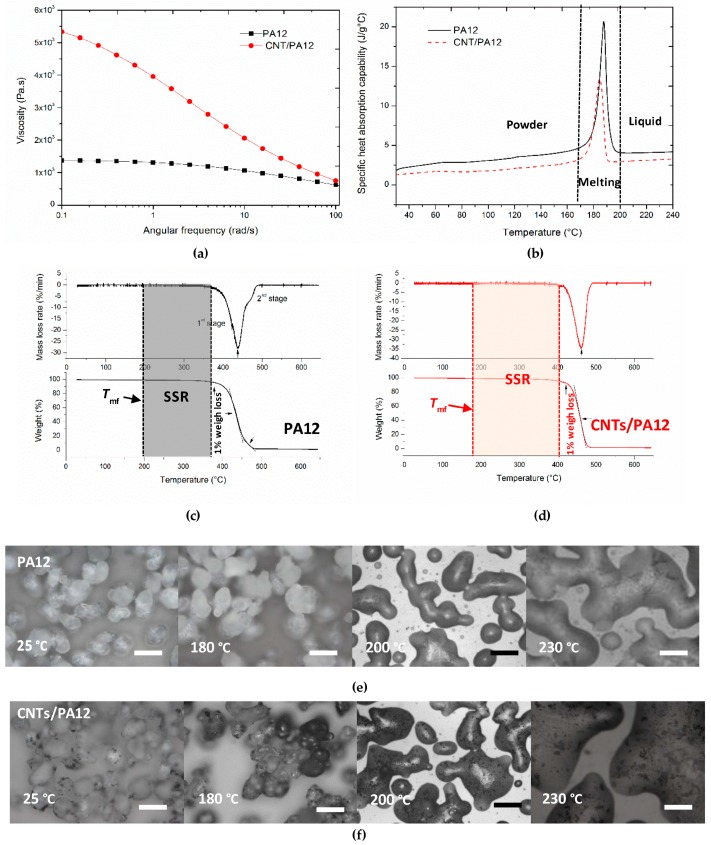
(**a**) melt viscosity of PA12 and CNTs/PA12 at 200 °C; (**b**) specific heat absorption of PA12 and CNTs/PA12 over the process temperature range; (**c**,**d**) TGA plots of decomposition processes of PA12 and CNTs/PA12, respectively; (**e**,**f**) the optical images of the fusion process of PA12 and PA12/CNTs powders (the scale bars are 100 µm).

The heat capacity is as a function of temperature, which is characterized through the modulated DSC approach [26,27]. CNTs/PA12 shows the lower heat capacity than PA12 in both powder and liquid phases. In Figure 3, the heat capacity of powders exhibits a positively linear relationship with temperature, whereas the heat capacity of liquid phase is almost remained as a constant for both neat PA12 and its composite. The linear data fittings are applied for melting and decomposition energy evaluations in Section 3.2. The modified heat capacities over the three stages of the powder, melting and liquid states are illustrated in Figure 4b, and this plot demonstrates the net-effect of temperature change and phase transitions on the modified specific heat corresponding to the process temperature range.

The proper fusion of powder particles is crucial in order to obtain fully condensed parts through laser sintering. The viscous sintering of polymer powders was described by the simplified model [18,28,29]

(13) $$\frac{x^{2}}{R}=\frac{2\Gamma }{3\eta_{0}}t$$

where x and R are respectively necking and particle radius, Γ and $\eta_{0}$ are the surface tension and zero-shear viscosity of the polymer melt and t is sintering time. Although this simple model is limited to perfectly spherical powders at the initial stage of sintering, the extended models [29] reveal that the shear viscosity is critical for judging material sinterability as compared with the surface tension, which is only a weak function of temperature.

The investigation into melt viscosity of polymer and its composite was applied to analyze the post-condensation and coalescence behaviors in laser sintering. The advantage of using melt viscosity to evaluate these behaviors, rather than direct molecular weight measurements such as gel permeation chromatography, is that the changes in molecular weight and influences of additives as a function of time and temperature can be directly determined [21]. The oscillatory measurements were conducted to obtain the melt viscosity of PA12 and CNTs/PA12 through varying the frequency from 0.1 to 100 rad/s (Figure 4a). As the typical polymers are shear-thinning materials, viscosities decrease as the applied angular frequency increases above a certain rate. However, as shown in Figure 4, the melt viscosity of PA12 is not sensitive to frequency change and a plateau phenomenon was observed at the low frequency range (<1 rad/s). Alternatively, the melt viscosity of CNTs/PA12 greatly decreases as the angular frequency increases. The composites exhibited the high melt viscosity at the low frequency range, especially at zero frequency, compared with the neat PA12. Such observations indicated that CNTs/PA12 may require more time to fuse and coalesce and even the laser could fully melt the powders. Therefore, the powder fusion under the pressure free condition was captured through an optical microscopy to observe the powder coalescence of PA12 and CNTs/PA12 (Figure 4e,f). CNTs/PA12 started to fuse at 180 °C but PA12 still retained the solid state. When the temperature increased above the melting point of powders, these powders were melted and gradually merged together. Fortunately, although the melt viscosity of CNTs/PA12 is higher than that of PA12 at the pressure free condition, the composite powders are able to fuse easily when they are fully melted at the elevated temperature. Thus the addition of CNTs with 0.5 wt % did not cause adverse effects during the powder coalescence.

To fix the stable sintering region (SSR) of laser sintering, the DSC and TGA analysis were combined to determine the offset of melting and onset of decomposition of materials. Usually, a polymer with a wide SSR via optimum temperature range is preferable for the laser sintering process. As shown in Figure 4c,d, CNTs/PA12 exhibited a wider range of SSR than PA12, and this observation indicated that the stable sintering was easily controlled and the decomposition of polymer composite could be prohibited even if the local temperature increases dramatically. Meanwhile, the temperature range of SSR could be implemented to quantitatively predict the energy required before decomposition through Equation (10).

**Figure 5:**
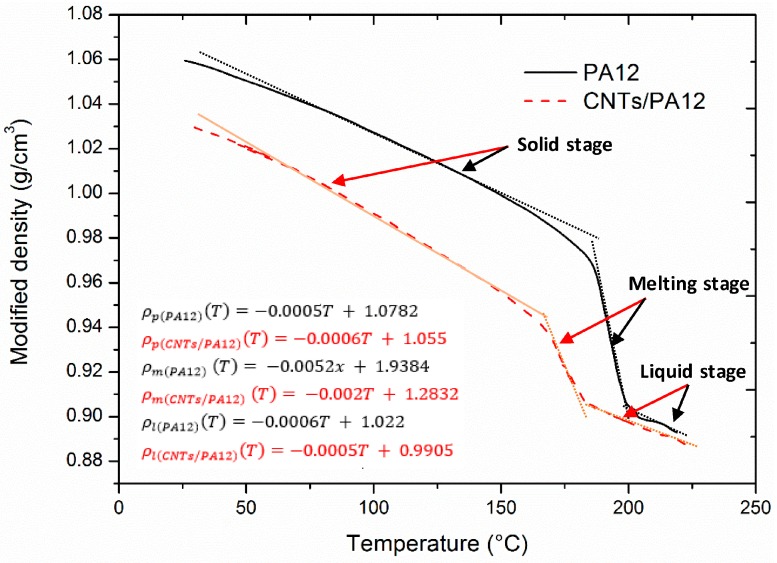
Increasing temperature from 25 to 225 °C, the modified densities of PA12 and CNTs/PA12 exhibit linear relationships with temperature in three stages via the solid, melting and liquid state.

Conventionally, the dilatometric properties were measured to investigate the potential of warping and shrinkage of the sintered parts, and the specific volume of the polymers behaves as a function of temperature upon the heating-cooling process [21]. In this work, in order to exactly predict the energy required for melting and decomposition, the reciprocal of specific volume was applied to evaluate the modified density, which was introduced to describe the trends of density change of PA12 and CNTs/PA12 upon heating. Figure 5 plotted the linear functions of densities in the three stages of materials used in laser sintering.

The thermal behaviors relevant to the sintering process were summarized in Table 3. The majority of temperature dependent properties were characterized and discussed. Packing factor (ϕ) represents the effectiveness of powders occupying a specific volume in the deposition process. The detailed measurement procedures were reported elsewhere [29] and packing factors of PA12 and CNTs/PA12 powders were evaluated by Peyre et al. [25]. The experimental measurements conducted by Bai et al. revealed that the thermal conductivities of PA12 and CNTs/PA12 powders were the weak function of temperature, and the influences of temperature on thermal conductivities were negligible in the theoretical evaluation [19].

### 3.2. Enhancements of CNTs in PA12 Matrix

The CNT-coating was able to enhance the PA12 matrix in two aspects: (i) the facilitation of heat absorption and conduction during laser sintering; and (ii) the reinforcement on mechanical properties of the sintered polymeric composites. The experimental observations and theoretical prediction were discussed as follows.

Using the experimental-theoretical approach, the energy required for melting per mass or per volume were evaluated and listed in Table 4. The value of *E*_mv_ of PA12 agreed with the prediction in the method proposed by Vasquez et al. [22], and the evaluation of *E*_dv_ was slightly higher than that reported previously due to the fact that the wide SSR was obtained in the current work (Table 4).

Whereas, the theoretical analysis shows that CNTs/PA12 required less energy to melt and consumed more energy till decomposition than PA12. This evaluation reveals that CNTs assist the polymer melting and result in ease of sintering, which can improve the effectiveness of laser sintering. Meanwhile, the improved thermal conductivity of composite powders also positively accelerates the thermal conduction within the polymer matrix and increases the penetration length of laser source through the powder bed [28], possibly resulting in the full melting of polymeric powders and the minimization of interlayer delamination.

**Figure 6:**
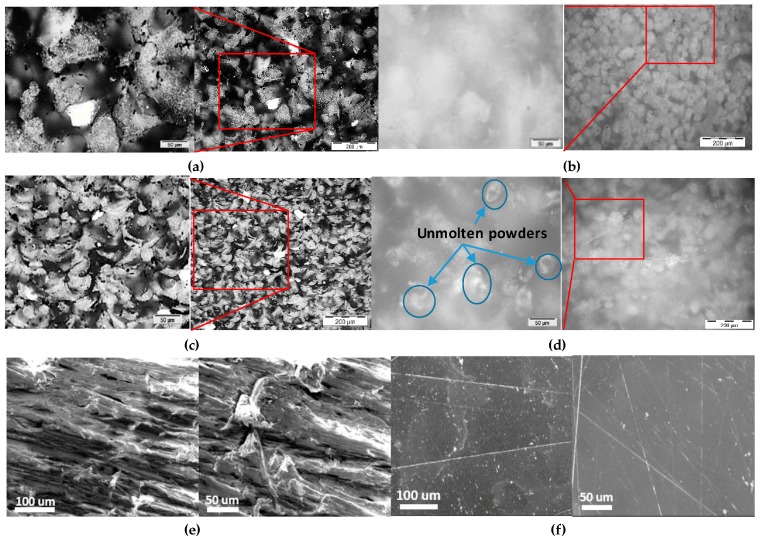
Optical images of microstructures of (**a**) CNTs/PA12 and (**b**) PA12 from the *X*–*Y* plane; (**c**) CNTs/PA12 and (**d**) PA12 specimen cross-section from the *X*–*Z* plane and the molten powders highlighted. SEM images of surface structures of (**e**) CNTs/PA12 and (**f**) PA12 through mechanical grinding and polishing.

Figure 6 shows the microstructures of the sintered PA12 and CNTs/PA12 from the *X*–*Y* and *X*–*Z* cross-sections. It is observed that CNT-coating still remained on the powder boundaries and formed a secondary phase to be embedded into the polymer matrix. The composite powders were fully sintered with random shapes on the *X*–*Y* plane, while the composite powders were melted and formed lots of menisci, which were condensed stacking together observed from the *X*–*Z* plane (Figure 6c). This indicated that the composite powders were fully melted and the molten polymer tended to spread within the confided space, and the formation of menisci is driven by gravity and surface tension at the liquid state. However, in Figure 6b,d, the PA12 powders still remained semi-spherical in shape and non-molten polymers were also shown on the *X*–*Z* cross-section. This reveals that PA12 powders were just partially melted to sinter and exhibited a weak tendency to fully fuse together. Although composites exhibited increased melt viscosity with limited melt flow capability compared with neat polymer (Figure 4a), the improved state of melting enabled enhancement of the coalescence and fusion of composite powders. In another words, the partially melted state of virgin polymer powders induces inferior melt flowability and adversely coalescence of powders. The surface morphologies show the cross-section of sintered CNTs/PA12 and PA12 after sand grinding; the composite exhibits very rough surface due to that CNT bundles were bonded with polymer and too stiff to remove. In comparison, the surface of grinding PA12 was smooth and flat (Figure 6e,f).

The relationship of melting and degradation energy with laser energy (Equation (12)) was applied to evaluate the heat absorption and predict the proper range of energy input by laser expressed as: $E_{mv}<E_{vol\;}\alpha_{critical}<E_{dv}$, where $\alpha_{critical\;}E_{vol\;critical}$ is total energy absorbed to fully melt polymer powders upon laser sintering. The greater value of $\alpha_{critical}$ reveals that more heat is absorbed when specific powders are exposed to laser scanning. $E_{vol\;critical}$ is the minimum value of laser energy input to fully melt this type of powders. Grewell et al. investigated that a maximum of 20% of infra-red laser energy was possibly absorbed by neat polyamides [30], therefore, the minimum energy input from laser $E_{vol}\;$possibly fully melt PA12 powders starting with a value of around 0.356 J/mm^3^ ($E_{mv}/20\%$). As per the set of laser parameters listed in Table 1, the energy input of laser $E_{vol}\;\mathrm{is}$ 0.333 J/mm^3^ corresponding to the sintered structures of PA12 and CNTs/PA12 in Figure 6. The ratios of $E_{mv}/E_{vol}$ for PA12 and CNTs/PA12 are respectively 19.4% and 21.8%. Based on the observations of microstructure characterization, it can be concluded that the $\alpha_{critical\left(CNTs/PA12\right)}$ is higher than the value of 0.218 but $\alpha_{critical\left(PA12\right)}$ is much lower than the value of 0.194 due to the fact that a large amount of partially melted PA12 powders is observed. This reveals that the portion of laser energy absorbed by PA12 is much less than 20% in the SLS process because light transmission, reflection and scattering of powders are strongly dissipated by laser energy.

**Figure 7:**
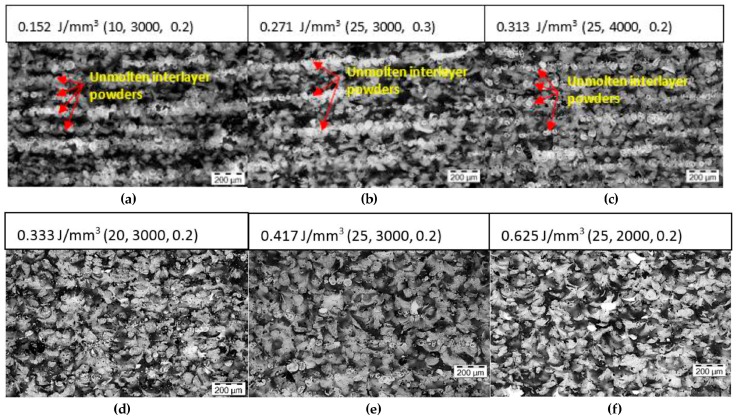
The microstructures of the CNTs/PA12 parts sintered upon varied laser input energy in the *X*–*Z* plane (power (W), scanning speed (mm/s), hatching space (mm)) (**a**–**f**).

The microstructures of sintered CNTs/PA12 parts under the different sets of laser energy were characterized from the *X*–*Z* plane to investigate the critical heat absorptivity $\alpha_{critical}$. As observed in Figure 7, the critical energy input was between 0.313 and 0.333 J/mm^3^ because the unmolten interlayer powders indicate the state of heat absorption by CNTs/PA12. Thus, the $\alpha_{critical}=E_{mv}/E_{vol\;critical}$ is around 0.22, revealing that 22% of laser energy input is absorbed by composite powders to effectively melt and sinter polymer.

**Figure 8:**
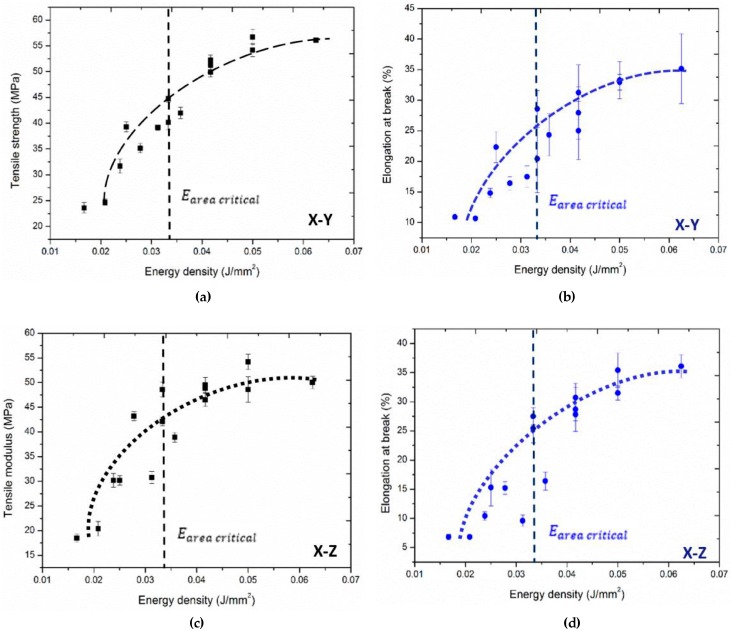
Energy density influences on (**a**) tensile strength and (**b**) elongation at break of specimens in the *X*–*Y* plane; (**c**) tensile strength and (**d**) elongation at break of specimens in the *X*–*Z* plane.

CNTs-coated PA12 is within the extended stable sintering region, resulting in the increased $E_{dv}$, and then $E_{vol}=E_{dv}/\alpha_{critical}$ could be estimated around 2.75 J/mm^3^ which is the energy required to induce polymer decomposition during the sintering process of CNTs/PA12. While, such energy density of laser is extremely high and rarely used in commercially available SLS machines. As shown in Figure 8, the mechanical properties tend to saturate once polymer powders are fully melted and coalesced before being decomposed. Thus, PA12 decomposition is not a critical issue causing inferior performances, the insufficient melting induced voids or interlayer delamination are more essential for improving the mechanical properties of materials.

Figure 8 shows that the tensile modulus and elongation at break of sintered CNTs/PA12 specimens in both of the *X*–*Y* and *X*-*Z* planes are tending to saturate when the laser energy input is above the crucial energy per volume $E_{vol\;critical}$, which is the minimum energy required to fully melt the composite powders as evaluated above. However, if the laser energy input is lower than $E_{vol\;critical}$, the mechanical performances drop significantly due to the insufficient melting as shown in Figure 7.

Another type of enhancements of CNTs on the mechanical aspect are as shown in Table 5, whereby the tensile strength and elongation at break both increased by 31.8% and 37.54%, respectively, compared with the neat PA12. Impressively, the toughness increased by 84.9% and the tensile moduli remained the same. In comparison, the ball-milled CNTs/PA12 composite powders for laser sintering were reported to increase the ultimate strength and flexural modulus by 9.3% and 31.5%, respectively, but the elongation at break dropped by 18% [31]. The glass and ceramic fillers were also introduced to improve the mechanical stiffness of sintered polymeric composite but sacrificed ductility under tension [32,33,34,35]. It is known that the critical shortcoming of sintered polymer composites is poor ductility due to the weak interfacial adhesion and micro-defects induced by fillers.

Whereas, the results of sintered CNTs-coated PA12 parts reveal that nanofillers simultaneously enhance the strength and ductility of sintered polymeric composite. As observed in Figure 7, the CNT-coated layer still remained at the powder boundary and was well embedded within the PA12 matrix after sintering. As a secondary phase in PA12 composite, the CNTs’ framework enables strengthening the polymer matrix and preventing intermolecular movement of polymer chains under loading. Meanwhile, the nanofillers via CNTs strongly adhere to the powder surface through a facilitated latex technique and then fuse with polymer chains upon melting-solidification in the sintering process. Thus, the interfacial defects are minimized and few voids are observed in the fully sintered CNTs/PA12 in Figure 6 and Figure 7. This reveals the importance of the process–structure–property relationship in laser sintering. Accordingly, it was found that the CNTs facilitate heat absorption and allow the polymer powders to fully melt and fuse effectively within a short time domain, and CNT fillers can remain at the powder boundary and form a 3D network within the matrix due to the pressure free and superfast heating-cooling process unique to laser sintering. Such a strengthening mechanism is helpful to overcome the limited ductility of laser-sintered parts and improve their strength and toughness.

**Figure 9:**
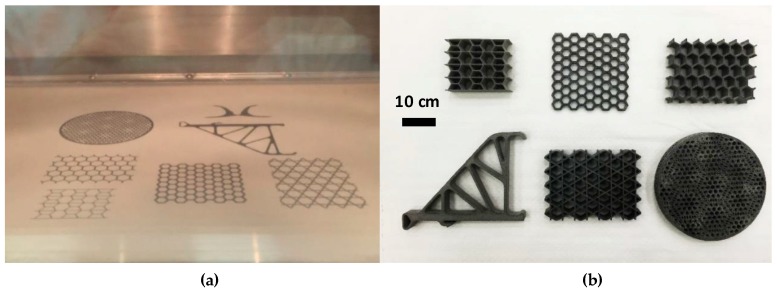
(**a**) The building platform of EOS P395 with the dimension of *X*, *Y*, *Z* (34 cm × 34 cm × 60 cm); (**b**) Light-weight CNTs/PA12 parts with complex geometric manufactured by SLS.

The optimized set of parameters (*p*: 25 W, *s*: 2500 mm/s, *h*: 0.2 mm and *L*: 0.1 mm) were successfully applied to print the three dimensional (3D) complex lightweight structures by CNTs/PA12 composites. These truss, honeycomb and kagome lattices, which are widely used lightweight engineering designs, were demonstrated in Figure 9. This reveals that the powder manufacturing process could supply a large quantity of powders used in the SLS system. The composite powder deposition and sintering are not barriers to produce the complex 3D structures in the full dimension of the machine building platform.

## 4. Conclusions

This work conducted a systematic method of powder evaluation and process optimization for newly developed composite powders. The intrinsic properties of new powders were characterized to predict the sintering behaviors and narrow the effective working range of laser parameters. Then, the microstructure and mechanical properties of simple specimens were tested to evaluate the state of sintering and optimize the set of parameters used in the process. Meanwhile, the optimized process parameters were applied to produce the 3D structure with a specific design for industrial applications. The unique 3D-framework of CNTs was established through laser sintering, and such a configuration and distribution of CNTs in the polymer matrix were found to strongly enhance its mechanical strength and toughness while simultaneously improving the elongation at break. 

## Figures and Tables

**Table 1:** SLS (selective laser sintering) parameters for PA12 and CNTs/PA12 powders.

Process parameter	Value
Laser power *p*, (W)	40
Scanning speed s, (mm/s)	4000
Hatching spacing *h*, (mm)	0.3
Bed temperature *T*_b_, (°C)	174
Chamber temperature *T*_c_, (°C)	130
Layer thickness *L*, (μm)	100
Laser beam diameter *d*, (mm)	0.42

**Table 2:** The sets of process parameters for CNTs/PA12 laser sintering.

Set	Fixed parameters	Controlled parameters					
1	*s*: 3000 (mm/s), *h*: 0.2 (mm)	*p* (W)	10	15	20	25	30
2	*p*: 25 (W), *h*: 0.2 (mm)	s (mm/s)	2000	2500	3000	3500	4000
3	*p*: 25 (W), *s*: 3000 (mm/s)	*h* (mm)	0.2	0.25	0.3	0.35	0.4

**Table 3:** Material parameters for PA12 and CNTs/PA12 powders.

Parameters	PA12	CNTs/PA12
Powder specific heat $C_{p}^{p}$, (J/g·°C)	Figure 3a	Figure 3a
Melt specific heat $C_{p}^{m}$, (J/g·°C)	Figure 3b	Figure 3b
Melting peak *T_m_*, (°C)	187.66	184.46
Enthalpy of melting ∆*H*_m_, (J/g)	107.2	90.85
Recrystallization peak *T*_r_, (°C)	150.90	156.22
Enthalpy of recrystallization ∆*H*_r_, (J/g)	50.04	46.11
Modified density $\rho^{*}$, (g/cm^3^)	Figure 5	Figure 5
Packing fraction ϕ	0.445	0.44
Thermal conductivity *K_p_*, (W/mK)	0.12 (±0.04)	0.25 (±0.08)
Onset melting temperature *T*_ms_, (°C)	180.93	177.21
Onset recrystallization temperature *T*_rs_, (°C)	155.70	159.22
Glass window width (GW), (°C)	25.23	17.99
Stable sintering region (SSR), (°C)	198–360	187–402
Melt viscosity at 0.1 rad/s,$\;\eta_{0}$, (Pa·s)	1.4 × 10^3^	5.4 × 10^3^

**Table 4:** Evaluation of energy required for melting and energy absorbed before polymer degradation with respect to the specific mass and volume; the measured thermal conductivities of PA12 and CNTs/PA12 parts.

Material features	PA12	CNTs/PA12
Mass energy for melt *E*_mm_, (J/g)	189.51	167.07
Volume energy for melt *E*_mv_, (J/mm^3^)	0.072	0.064
Mass energy before decomposition *E*_dm_, (J/g)	688.96	767.52
Volume energy before decomposition *E*_dv_, (J/mm^3^)	0.555	0.606
Thermal conductivity *K*_o_, (W/mK)	0.15 (±0.05)	0.45 (±0.09)

**Table 5:** Comparison of the mechanical properties of laser sintered PA12 and CNTs/PA12 parts.

Properties	Tensile modulus (MPa)	Tensile strength (MPa)	Elongation at break (%)	Toughness (MJ/mm^3^)
PA12	1291 (±12.1)	44 (±1.3)	24 (±0.8)	10.18 (±0.9)
CNTs/PA12	1301 (±14.5)	58 (±2.4)	33 (±2.4)	18.86 (±1.8)
Enhancement (%)	0.8	31.8	37.5	84.9

## References

[1] Chua C.K., Leong K.F. (2014). 3D Printing and Additive Manufacturing: Principles and Applications.

[2] Aliakbari M. (2012). Additive Manufacturing: State-of-the-Art, Capabilities, and Sample Applications with Cost Analysis. Master’s Thesis.

[3] (2010). ASTM F2792. Standard Terminology for Additive Manufacturing Technologies.

[4] Goodridge R.D., Tuck C.J., Hague R.J.M. (2012). Laser sintering of polyamides and other polymers. Prog. Mater. Sci..

[5] Wen S.F., Yan C.Z., Wei Q.S., Zhang L.C., Zhao X., Zhu W., Shi Y.S. (2014). Investigation and development of large-scale equipment and high performance materials for powder bed laser fusion additive manufacturing. Virtual Phys. Prototyp..

[6] Papadakis L., Loizou A., Risse J., Bremen S., Schrage J. (2014). A computational reduction model for appraising structural effects in selective laser melting manufacturing. Virtual Phys. Prototyp..

[7] Wu W., Tor S.B., Chua C.K., Leong K.F., Merchant A. (2015). Investigation on processing of ASTM A131 Eh36 high tensile strength steel using selective laser melting. Virtual Phys. Prototyp..

[8] Yadroitsev I., Yadroitsava I. (2015). Evaluation of residual stress in stainless steel 316L and Ti6Al4V samples produced by selective laser melting. Virtual Phys. Prototyp..

[9] Park S., Lim T., Yang D.-Y., Kim R., Lee K.-S. (2006). Improvement of spatial resolution in nano-stereolithography using radical quencher. Macromol. Res..

[10] Jariwala A.S., Ding F., Boddapati A., Breedveld V., Grover M.A., Henderson C.L., Rosen D.W. (2011). Modeling effects of oxygen inhibition in mask-based stereolithography. Rapid Prototyp. J..

[11] Kumar K., Kumar G.S. (2015). An experimental and theoretical investigation of surface roughness of poly-jet printed parts. Virtual Phys. Prototyp..

[12] Versavaud S., Régnier G., Gouadec G., Vincent M. (2014). Influence of injection molding on the electrical properties of polyamide 12 filled with multi-walled carbon nanotubes. Polymer.

[13] Zhu W., Yan C., Shi Y., Wen S., Liu J., Shi Y. (2015). Investigation into mechanical and microstructural properties of polypropylene manufactured by selective laser sintering in comparison with injection molding counterparts. Mater. Des..

[14] Song K., Zhang Y., Meng J., Green E., Tajaddod N., Li H., Minus M.L. (2013). Structural polymer-based carbon nanotube composite fibers: Understanding the processing–structure–performance relationship. Materials.

[15] Meyer K.R., Hornung K.H., Feldmann R., Smigerski H.J. (1982). Method for Polytropically Precipitating Polyamide Powder Coating Compositions Where the Polyamides Have at Least 10 Aliphatically Bound Carbon Atoms Per Carbonamide Group. U.S. Patent.

[16] Zhang Y., Song K., Meng J., Minus M.L. (2013). Tailoring polyacrylonitrile interfacial morphological structure by crystallization in the presence of single-wall carbon nanotubes. ACS Appl. Mater. Interfaces.

[17] Bai J., Yuan S., Chow W., Chua C.K., Zhou K., Wei J. (2015). Effect of surface orientation on the tribological properties of laser sintered polyamide 12. Polym. Test..

[18] Bai J.M., Goodridge R.D., Hague R.J.M., Song M. (2013). Improving the mechanical properties of laser-sintered polyamide 12 through incorporation of carbon nanotubes. Polym. Eng. Sci..

[19] Bai J., Goodridge R., Yuan S., Zhou K., Chua C., Wei J. (2015). Thermal influence of CNT on the polyamide 12 nanocomposite for selective laser sintering. Molecules.

[20] Berretta S., Evans K.E., Ghita O. (2016). Predicting processing parameters in high temperature laser sintering (HT-LS) from powder properties. Mater. Des..

[21] Verbelen L., Dadbakhsh S., Van den Eynde M., Kruth J.-P., Goderis B., Van Puyvelde P. (2016). Characterization of polyamide powders for determination of laser sintering processability. Eur. Polym. J..

[22] Vasquez M., Haworth B., Hopkinson N. (2013). Methods for quantifying the stable sintering region in laser sintered polyamide-12. Polym. Eng. Sci..

[23] Cai D., Jin J., Song M. (2010). Process. U.S. Patents.

[24] Yuan S., Bai J., Chua C.K., Zhou K., Jun W. (2016). Highly enhanced thermal conductivity of thermoplastic nanocomposites with a low mass fraction of MWCNTs by a facilitated latex approach. Compos. Part A Appl. Sci. Manuf..

[25] Scholten H., Christoph W. (2001). Use of a Nylon-12 for Selective Laser Sintering. U.S. Patent.

[26] Danley R.L. (2003). New heat flux DSC measurement technique. Thermochim. Acta.

[27] Peyre P., Rouchausse Y., Defauchy D., Régnier G. (2015). Experimental and numerical analysis of the selective laser sintering (SLS) of PA12 and PEKK semi-crystalline polymers. J. Mater. Process. Technol..

[28] Bai J., Goodridge R.D., Hague R.J.M., Song M., Okamoto M. (2014). Influence of carbon nanotubes on the rheology and dynamic mechanical properties of polyamide-12 for laser sintering. Polym. Test..

[29] Ristić M.M., Milosević S. (2006). Frenkel’s theory of sintering. Sci. Sinter..

[30] Grewell D., Rooney P., Kagan V.A. (2004). Relationship between optical properties and optimized processing parameters for through-transmission laser welding of thermoplastics. J. Reinf. Plast. Compos..

[31] Salmoria G.V., Paggi R.A., Lago A., Beal V.E. (2011). Microstructural and mechanical characterization of PA12/MWCNTs nanocomposite manufactured by selective laser sintering. Polym. Test..

[32] Chung H., Das S. (2006). Processing and properties of glass bead particulate-filled functionally graded Nylon-11 composites produced by selective laser sintering. Mater. Sci. Eng. A.

[33] Lee G. (1997). Selective Laser Sintering of Calcium Phosphate Materials for Orthopedic Implants. Ph.D. Thesis.

[34] Tan W.S., Chua C.K., Chong T.H., Fane A.G., Jia A. (2016). 3D printing by selective laser sintering of polypropylene feed channel spacers for spiral wound membrane modules for the water industry. Virtual Phys. Prototyp..

[35] Francis V., Jain P.K. (2016). Experimental investigations on fused deposition modelling of polymer-layered silicate nanocomposite. Virtual Phys. Prototyp..

